# Concurrent predictors of word reading and reading comprehension for 9-year-olds with Williams syndrome

**DOI:** 10.1007/s11145-021-10163-4

**Published:** 2021-07-03

**Authors:** Carolyn B. Mervis, Caroline Greiner de Magalhães, Cláudia Cardoso-Martins

**Affiliations:** 1 Department of Psychological and Brain Sciences, University of Louisville, 317 Life Sciences Building, Louisville, KY 40292 USA; 2 Department of Psychology, Universidade Federal de Minas Gerais, Belo Horizonte, MG Brazil

**Keywords:** Williams syndrome, Word reading, Reading comprehension, Phonological skills, Reading instruction method, Intellectual disability

## Abstract

We examined the cognitive, language, and instructional factors associated with reading ability in Williams syndrome (WS). Seventy 9-year-olds with WS completed standardized measures of real-word reading, pseudoword decoding, reading comprehension, phonological skills, listening comprehension, nonverbal reasoning, visual-spatial ability, verbal working memory, rapid naming, and vocabulary. Reading instruction method was determined from school records and interviews with parents and teachers. Similar to prior findings for individuals with WS, reading ability varied widely, ranging from inability to read any words to reading comprehension at age level. Multiple regression analyses indicated that the primary concurrent predictor of word reading ability was reading instruction method, with a systematic phonics approach associated with considerably better performance than other reading instruction approaches. Phonological processing skills—as assessed by a composite of phonological awareness and verbal short-term memory—also contributed significant unique variance to word reading ability, as did visual-spatial ability. The concurrent predictors of reading comprehension were single-word reading and listening comprehension. These findings indicate that the factors that predict concurrent early word reading and reading comprehension abilities for children with WS are consistent with previous findings for typically developing children and that the Simple View of Reading applies to children with WS. Children with WS benefit strongly from systematic phonics instruction regardless of IQ. Instruction focused on improving listening comprehension is likely to improve reading comprehension, especially as word reading skills increase.

**Supplementary Information:**

The online version contains supplementary material available at 10.1007/s11145-021-10163-4.

## Introduction

The ability to read is critical for acquisition of knowledge, success in the workplace, and access to leisure activities, all of which contribute to quality of life and mental health (Castles et al., 2018; National Reading Panel, 2000). For individuals with intellectual disability (ID), difficulty with reading is both the most frequently identified secondary (preventable or ameliorable) condition and the one their caregivers consider to result in the greatest limitation (Koritsas & Iacono, 2011). Findings from a recent survey of 211 parents of school-aged children with ID (Wakeman et al., 2021) indicated that 93% considered it very important for their child to learn to read and 85% thought that children who were successful in learning to read would have better life outcomes than children who were not.

Reading difficulty is very common among individuals with Williams syndrome (WS), a genetic disorder associated with mild to moderate ID. Many older teenagers and adults with WS are not able to read at all (Brawn et al., 2018; Howlin et al., 1998), and even after those individuals were excluded, average single-word reading age equivalents (AEs) for high school students or adults were in the 8-year range. These findings suggest that at the end of formal schooling, the reading abilities of individuals with WS typically are below the level of functional literacy. At the same time, several studies of individuals with WS have identified one or more individuals who would be considered very good readers. For example, in the first study addressing the reading abilities of individuals with WS, Pagon and colleagues (1987) reported that a participant in grade 9 performed at grade level on standardized tests of both single-word reading and reading comprehension. Mervis (2009) indicated that the highest-performing student in her sample earned standard scores (SSs) above 100 on single-word reading, pseudoword decoding, and reading comprehension. Levy and Antebi (2004), who considered only single-word reading, reported that one 19-year-old participant read at age level. Given these stark contrasts, identification of the language, cognitive, and instructional characteristics associated with variability in word reading and reading comprehension ability among individuals with WS is vital.

The purpose of this study was to document individual differences in the early reading abilities of a substantial sample of 9-year-old children with WS and to determine if the same characteristics that have been associated with variations in reading ability among typically developing (TD) children also are concurrent predictors of reading ability in children with WS. In the remainder of the Introduction we briefly review the literature addressing characteristics that have been found to influence the reading abilities of TD children. We then consider prior studies of the reading abilities of individuals with WS and describe the goals of the present study.

### Factors affecting reading skills of typically developing children

#### Word reading

It is well established that phonological processing skills (the ability to access, store, and manipulate speech sounds) are among the strongest predictors of reading ability in an alphabetic orthography (Melby-Lervåg et al., 2012; Wagner et al., 2019). Together with knowledge of grapheme-phoneme correspondences, these processes form the basis of readers’ ability to read by phonological recoding or decoding, that is, by translating letters or units of letters into the sounds they represent in words and then blending the sounds to derive the word’s pronunciation, an ability at the heart of skilled reading (e.g., Ehri, 2005; Share, 1995).

Of the phonological processes studied in connection to word reading, phoneme awareness (the ability to consciously attend to and manipulate phonemes) is most strongly associated with word reading accuracy (Melby-Lervåg et al., 2012; Wagner et al., 2019). Phoneme awareness is crucial for cracking the alphabetic code and reading by phonological recoding, but this ability does not develop spontaneously (Morais et al., 1979). In natural speech, phonemes run together, making it difficult to pinpoint where one ends and the next begins. Systematic phonics instruction, which teaches children to segment words into their constituent phonemes and to associate these phonemes with specific graphemes, allows children to decode by translating printed words into their spoken counterparts based on grapheme-phoneme connections. This type of instruction has been shown repeatedly to result in not only stronger decoding skills but also better reading comprehension than nonsystematic phonics instruction or instruction that does not include any phonics (Castles et al., 2018; Moats, 2019; National Reading Panel, 2000; Rose, 2006).

In addition to phoneme awareness, broader language skills, including vocabulary, morphology, and grammar, have sometimes been linked to word reading ability (Nation & Snowling, 2004), as have several domain general processes, particularly verbal working memory (see Peng et al., 2018 for a meta-analysis) and nonverbal reasoning (Dolean et al., 2019). Although less often considered, visual-spatial ability also has been found to be related to word reading accuracy for TD children (Greiner de Magalhães et al., 2021) and visual-spatial attention deficits have been reported for children at familial risk for reading disability (Facoetti et al., 2010). However, relative to phoneme awareness, the direct contribution of these factors is small. A second phonological process—phonological recoding in lexical access (rapid naming), which allows for rapid and accurate retrieval of phonological codes from long-term memory—although sometimes related to word reading accuracy, is more strongly associated with word reading fluency (Lervåg & Hulme, 2009).

#### Reading comprehension

The ultimate goal of reading is comprehension. According to the Simple View of Reading (Gough & Tunmer, 1986), reading comprehension is the product of decoding and listening comprehension. A logical consequence of Gough and Tunmer’s formula is that both decoding and listening comprehension are necessary for reading comprehension. When children are learning to decode, their ability to understand what they read will be largely constrained by limitations in decoding skills. Conversely, as decoding skills increase listening comprehension becomes a more important predictor of reading comprehension. There is considerable empirical evidence supporting the Simple View of Reading for TD children (Language and Reading Research Consortium & Chiu, 2018; Quinn & Wagner, 2018). Nonverbal reasoning also has been associated with reading comprehension (Lervåg et al., 2019).

#### Reading skills of individuals with Williams syndrome

WS is a rare neurodevelopmental disorder caused by a hemideletion of 25–27 genes on chromosome 7q11.23. The prevalence of WS is one in 7500 live births (Strømme et al., 2002). Individuals with WS typically have mild to moderate ID, although the full range of intellectual ability associated with this syndrome is from the bottom of the average range for the general population to severe ID. The WS cognitive phenotype is characterized by relative strengths in concrete language, nonverbal reasoning, verbal short-term memory, and phonological awareness, and considerable weakness in visual-spatial construction, receptive grammar, and relational/conceptual language (Mervis, 2009; Mervis & John, 2010). In a recent study investigating individual differences in the performance of 49 individuals with WS aged 6–39 years on the Woodcock-Johnson Tests of Cognitive Abilities-III (Woodcock et al., 2001), Miezah and colleagues (2020) found that, on average, participants performed best on the Phonemic Awareness cluster (mean SS = 94.36, *SD* = 21.03, range: 60–129). (Note that this cluster measures phonological awareness rather than phoneme awareness.) This finding suggests that a considerable proportion of individuals with WS have phonological processing abilities in the average range for the general population.

As discussed at the beginning of this article, there is considerable variability in the single-word reading abilities of individuals with WS. This variability, which has been documented not only for English (Brawn et al., 2018; Laing et al., 2001; Levy et al., 2003; Mervis, 2009; Pagon et al., 1987; Steele et al., 2013), but also for Italian (Menghini et al., 2004) and Hebrew (Levy & Antebi, 2004), is best illustrated by considering children’s SSs on reading assessments, which provide a measure of reading performance relative to same-aged peers in the general population. Mervis (2009) reported that for 44 children with WS aged 9–17 years, mean SS on the Wechsler Individual Achievement Test-II (WIAT-II; Wechsler, 2005) was 73.00 (range: 40 [lowest possible]–112) on the Word Reading subtest, 78.75 (range: 0 correct—113) on the Pseudoword Decoding subtest, and 64.61 (range: 40 [lowest possible]—102) on the Reading Comprehension subtest, with *SD*s > 15 on all three subtests.

Correlational analyses addressing relations between reading ability and the cognitive and language variables considered for TD children earlier in this paper also have been conducted for individuals with WS. Significant and strong relations between phonological awareness and word reading have been reported for English (Brawn et al., 2018; Laing et al., 2001; Levy et al., 2003; Steele et al., 2013), Italian (Menghini et al., 2004), and Hebrew (Levy & Antebi, 2004). In all but one of these studies (Steele et al., 2013), participants were adolescents or adults. Correlations between verbal short-term memory and single-word reading ranged from weak (Laing et al., 2001) to moderate (Brawn et al., 2018) to strong (Levy & Antebi, 2004). Relations between rapid naming and single-word reading ranged from very weak (Brawn et al., 2018; Levy & Antebi, 2004) to moderate (Levy et al., 2003) to strong (Laing et al., 2001).

Relations with other variables also have been considered. Correlations between vocabulary and single-word reading were moderate (Laing et al., 2001; Levy et al., 2003; Steele et al., 2013). Correlations with verbal ability were moderate (Brawn et al., 2018; Laing et al., 2001) to strong (Levy et al., 2003). In the one study that considered verbal working memory, a strong relation with single-word reading was found (Brawn et al., 2018). Correlations with nonverbal reasoning ability were mixed, ranging from weak (Levy et al., 2003) to moderate (Brawn et al., 2018) to strong (Laing et al., 2001). Correlations with visual-spatial ability were moderate (Brawn et al., 2018) to strong (Laing et al., 2001; see Dessalegn et al., 2013 for a case-study comparison). Correlations between single-word reading and overall IQ were moderate (Brawn et al., 2018) to strong (Laing et al., 2001). When participants were split into groups based on IQ, the higher-IQ group(s) had better single-word reading abilities than the lower-IQ group (Howlin et al., 1998; Levy et al., 2003; Udwin et al., 1987).

Reading comprehension was considered in three studies. Menghini et al. (2004) reported a moderate correlation between reading comprehension ability and mental age. Laing et al. (2001) reported that reading comprehension ability was very strongly correlated with single-word reading ability; strongly correlated with nonverbal reasoning ability, visual-spatial ability, listening comprehension, and overall IQ; and moderately correlated with vocabulary, verbal ability, and verbal short-term memory ability. Single-word reading was significantly stronger than reading comprehension in the two studies in which SSs were compared (Laing et al., 2001; Mervis, 2009). As has been repeatedly shown for TD children (e.g., Castles et al., 2018), Mervis (2009) reported that children who were taught to read using a systematic phonics approach had significantly higher SSs on both word reading and reading comprehension than did children taught to read with other approaches, even after taking into account differences in overall IQ. Findings from a recent meta-analysis of beginning reading interventions for children and adolescents with ID (Reichow et al., 2019) indicated that relative to instruction-as-usual, interventions including elements of phonological awareness, letter sound instruction, and decoding had a moderate positive effect on word reading.

### Current study

Given the importance of reading skills for both academic achievement and adaptive independence (Brawn et al., 2018), identification of factors that affect the reading abilities of individuals with WS is vital. Although prior studies have addressed this question, most of them have focused on comparisons of individuals with WS to considerably younger TD children matched for reading age or mental age (e.g., Laing et al., 2001; Menghini et al., 2004) and/or simple relations between raw scores or age equivalents on reading measures and potential correlates (e.g., Levy & Antebi, 2004; Levy et al., 2003). Interpretation of these findings is difficult due to psychometric problems involved in comparisons with much younger TD children matched for one ability (e.g., single-word reading raw score or AE), but almost certainly not matched for other relevant abilities (e.g., phonological processing, verbal, nonverbal reasoning, visual-spatial) (see Brawn et al., 2018; Mervis & Klein-Tasman, 2004 for a discussion of these problems) and psychometric problems with the use of AEs rather than SSs (Mervis & Klein-Tasman, 2004; Mervis & Robinson, 2005). Additional concerns include the limited sample size (15–30 participants) of the studies that investigated the correlates of reading in WS, wide age ranges—extending from childhood through adulthood in most studies—and/or the use of SSs from standardized assessments that are not normed for individuals as old as the participants.

In the present study, we focus on children with WS and consider major factors that have been identified in theoretical accounts of reading development and/or empirical studies of TD children or children with WS as contributing to individual differences in reading abilities. Our first goal is to document the variability among same-aged children with WS in both word reading (including both actual English words and pseudowords) and reading comprehension.

Our second goal is to determine if the factors that affect variation in word reading skills among TD children also are concurrent predictors of variation among children with WS. To address this goal, we considered seven potential current predictors: reading instruction method, phonological processing skills (as assessed by a composite of phonological awareness and verbal short-term memory), rapid naming, vocabulary, nonverbal reasoning ability, visual-spatial ability, and verbal working memory. Based on prior findings from studies of TD children or children with WS, we hypothesized that single-word reading ability would be concurrently predicted by reading instruction method, phonological skills, and visual-spatial ability.

Our third goal is to determine if the factors that affect variation in reading comprehension among TD children are concurrent predictors of variation in reading comprehension among children with WS. Based on prior findings from TD children or children with WS, we considered five potential predictors: single-word reading, listening comprehension, nonverbal reasoning ability, verbal working memory, and rapid naming. Based on the Simple View of Reading, we hypothesized that reading comprehension ability would be concurrently predicted by single-word reading ability and listening comprehension ability. Given the participants’ age and anticipated relatively limited single-word reading abilities, we expected that single-word reading ability would be a stronger predictor than listening comprehension ability.

To address these goals, we studied 70 9-year-olds with WS. This is the youngest age at which the first author’s lab administers academic achievement assessments to children with WS. All dependent variables and all continuous independent variables were assessed using age-appropriate standardized assessments, with performance measured by SSs or T-scores.

## Method

### Participants

The final sample included 70 children (34 girls, 36 boys) aged 9.01–9.89 years (*M* = 9.32, *SD* = 0.27). All had genetically-confirmed classic-length deletions of the WS region and were native speakers of English. Mean General Conceptual Ability (GCA, similar to IQ) on the Differential Ability Scales-II School Age version (DAS-II; Elliott, 2007) was 64.94 (*SD* = 11.58, range: 41–95). Participants lived in 23 different U. S. states (representing all U.S. census regions: 20.0% Northeast, 42.8% South, 24.3% Midwest, 10.0% West) and two Canadian provinces (2.9%). The distribution of participants’ racial/ethnic background was: 78.5% White non-Hispanic, 11.4% White Hispanic, 2.9% African-American non-Hispanic, 4.3% multiracial non-Hispanic, and 2.9% multiracial Hispanic. Nineteen of the participants’ mothers (27.1% of the final sample) did not have a bachelor degree; the remaining 51 (72.9%) had earned at least a bachelor degree. Some of the participants were enrolled in a longitudinal study. In that study, the youngest age at which academic achievement testing is included in the protocol is 9 years. Thus, the data reported for all participants are from the first (or only) session at which reading achievement was measured.

The participants’ median grade in school was 3rd, with an IQR from 2nd to 3rd grade and a range from the summer after 1st grade to 4th grade. Primary classroom placement was in a mainstream class for 47 children (17 with reading instruction primarily in the mainstream, 30 with reading instruction primarily in a resource room or other special education classroom) and in a special education (self-contained) class for 19 children (all with reading instruction in a special education classroom). The remaining 4 children were homeschooled.

Two additional 9-year-olds with genetically confirmed classic deletions were excluded, one because her standardized residual was > 3 SDs below the mean for the regression predicting single-word reading and one because he was nonverbal. Data collection began in December 2009 and ended in February 2020.

### Measures

#### Vocabulary ability

Vocabulary ability was measured by a composite based on the mean of each child’s SSs on the Peabody Picture Vocabulary Test-4 (PPVT-4; Dunn & Dunn, 2007) and the Expressive Vocabulary Test-2 (EVT-2; Williams, 2007). Based on the test manuals, split-half internal consistency for the 9-year-olds included in the norming sample was .90 for both the PPVT-4 and the EVT-2. For the present participants, the correlation between the PPVT-4 and EVT-2 SSs was *r* = .83, *p* < .001.

#### Nonverbal reasoning ability

Nonverbal reasoning was measured by the DAS-II Nonverbal Reasoning cluster SS. This cluster SS is based on performance on two subtests, one measuring matrix reasoning and one measuring sequential reasoning. Based on the test manual, IRT-based internal consistency for the 9-year-olds in the norming sample was .94.

#### Spatial ability

Spatial ability was measured by the DAS-II Spatial cluster SS. This cluster SS is based on performance on two subtests. The Pattern Construction subtest measures visual-perceptual matching (including spatial orientation) when copying block patterns. The Recall of Designs subtest measures short-term memory for visual-spatial relations (abstract patterns) as assessed by the child’s drawings of these patterns from memory. IRT-based internal consistency for the 9-year-olds in the DAS-II norming sample was .94.

#### Phonological skills

To measure phonological skills, a composite T-score based on performance on two DAS-II subtests was computed. The Phonological Processing subtest measures sensitivity to rhyme and the ability to blend, delete, and identify individual sounds in spoken English words. The Recall of Digits—Forward subtest measures short-term verbal recall of strings of digits produced by the examiner at a rate of two digits per second. For the participants in this study, the correlation between T-scores on these two subtests was *r* = .59, *p* < .001. IRT-based internal consistency for the 9-year-olds in the DAS-II norming sample was .87 for the Phonological Processing subtest and .93 for the Recall of Digits—Forward subtest. To form the phonological skills composite, the child’s T-scores for the two subtests were averaged.

#### Verbal working memory

Verbal working memory was measured by the DAS-II Recall of Digits—Backward subtest, which yields a T-score. This subtest measures the child’s ability to repeat digits in the reverse order from that presented by the examiner. Digits are presented at a rate of two per second. As reported in the DAS-II manual, IRT-based internal consistency for the 9-year-olds in the norming sample was .90.

#### Rapid naming

Rapid naming ability was measured by the T-score on the DAS-II Rapid Naming subtest, which measures speed and accuracy of naming the colors of colored squares, the names of black-and-white animals, and the colors and names of colored animals. All of the colors and animals used are common and were well known to the participants. For this subtest, IRT-based internal consistency for the 9-year-olds in the norming sample was .81.

#### Listening comprehension

Listening comprehension ability was measured by a composite SS based on performance on the Kaufman Brief Intelligence Test-2 (KBIT-2; Kaufman & Kaufman, 2004) Verbal scale and the Test for Reception of Grammar-2 (TROG-2; Bishop, 2003). The KBIT-2 Verbal scale includes two subscales, one measuring receptive vocabulary and general knowledge and one measuring verbal comprehension and reasoning. Split half internal consistency for the 9-year-olds in the norming sample was .91. The TROG-2 measures comprehension of 20 grammatical constructions ranging in difficulty from simple subject-verb sentences to sentences including center-embedded clauses. Split-half internal consistency as reported in the TROG-2 manual was .88 for the participants in the norming sample. For the participants in this study, the correlation between KBIT-2 Verbal SS and TROG-2 SS was *r* = .53, *p* < .001. To form the listening comprehension composite, the child’s KBIT-2 Verbal scale SS and TROG-2 SS were averaged.

#### Word reading

Word reading was measured by the Wechsler Individual Achievement Test-III (WIAT-III; Wechsler, 2009) Basic Reading Composite, which includes two subtests, one measuring single real-word reading (Word Reading) and one measuring single non-word reading (Pseudoword Decoding). According to the WIAT-III technical manual, split half internal consistency for the 9-year-olds in the norming sample was .98 for both Word Reading and Pseudoword Decoding and .99 for Basic Reading Composite. For the present participants, the correlation between Word Reading SS and Pseudoword Decoding SS was *r* = .87, *p* < .001.

#### Reading comprehension

Reading comprehension was assessed by the WIAT-III Reading Comprehension subtest, which measures comprehension of short passages read by the child. In accordance with the standardized instructions, the child was permitted to read the story either aloud or silently and reading errors were not corrected by the examiner. After the child finished reading a passage, the examiner asked 4–8 open-ended questions, depending on the passage. The passage remained in front of the child while he or she answered the questions. Split half internal consistency for the 9-year-olds in the WIAT-III norming sample was .80.

#### Reading instruction method

The primary approach to teaching reading to each child was classified as Systematic Phonics (hereafter, Phonics) or Other. All available information related to the students’ reading instruction was considered (e.g., reading program [if any] implemented in the primary classroom in which the child received reading instruction, Individualized Education Plan goals and progress reports, worksheets, homework assignments, conversations with parents and reading instructors). Reading instruction was classified as “Phonics” if the primary approach to teaching word reading was based on systematic instruction in English phonics. Reading instruction was classified as “Other” if it took a whole-language, three-cueing, or balanced literacy approach or otherwise emphasized the use of context to figure out a word or if it focused on whole-word instruction. The primary reading instruction approach was Phonics for 32 participants (46%) and Other for 38 participants (54%). Of the 32 children in the Phonics group, reading instruction took place primarily in their mainstream class for 14, in a self-contained class for 16, and in homeschool for 2. Of the 38 children in the Other group, reading instruction took place primarily in their mainstream class for 3, in a self-contained class for 33, and in homeschool for 2.

Mean chronological age, which was 9.34 years (*SD* = 0.26) for the Phonics group and 9.31 years (*SD* = 0.28) for the Other group, did not differ significantly, *t*(68) = 0.57, *p* = .570. A Mann–Whitney U test indicated that the distribution of grade (*Mdn* = 3rd grade, *IQR* = 2nd grade—3rd grade for each group) also did not differ significantly as a function of Reading Instruction Method group, *z* = 0.46, *p* = .645. However, for the children who were not homeschooled, the distribution of reading instruction method differed significantly as a function of the type of classroom in which reading instruction was provided, *χ*^2^ = 12.57, *p* < .001, with children whose reading instruction was in their mainstream class more likely to be in the Phonics group and children whose reading instruction was in a special education classroom more likely to be in the Other group. At the same time, it is important to note that the majority of the children in the Phonics group received most or all of their reading instruction in a special education classroom rather than in a mainstream classroom.

### Procedure

The study protocol was reviewed and approved by the university’s Institutional Review Board. Parents or legal guardians of all participants provided written informed consent and participants provided oral or written assent. Children completed the standardized measures at the first author’s laboratory as part of a larger two-day assessment. All measures were administered by trained doctoral students or research assistants and scored according to the standardized procedures.

## Results

Data were analyzed using IBM SPSS version 26.

### Performance on standardized assessments

Descriptive statistics for participants’ SSs or T-scores on the continuous variables included in the regression analyses are presented in Table 1. There was considerable variability, with scores on all measures ranging from average or above average for the general population to moderate-severe disability.

**Table 1:** Descriptive statistics for continuous variables included in the regression analyses

Variable	Mean	Median	SD	Range
Vocabulary SS	82.69	83.50	12.75	51–113
Nonverbal Reasoning SS	77.31	75.50	12.47	49–103
Spatial SS	55.04	54.50	14.09	32^a^–90
Phonological skills T	39.51	41.50	9.59	10–54
Rapid Naming T	36.94	38.00	7.34	11–51
Verbal working memory T	29.60	33.00	12.27	10–50
Listening comprehension SS	76.09	74.25	14.07	51–113
Basic Reading Composite SS	73.74	73.00	12.71	52^b^–106
Reading Comprehension SS	68.23	69.00	18.09	40^a^–110

*N* = 70. SS = standard score; T = T-score

^a^Lowest possible standard score

^b^Lowest possible standard score for older 9-year-olds

As indicated in the Introduction, there are serious psychometric concerns regarding AE scores (e.g., Mervis & Klein-Tasman, 2004; Mervis & Robinson, 2005). However, as AEs are the only statistical measure provided in many of the prior studies of the reading abilities of individuals with WS, nonparametric descriptive statistics for the WIAT-III Word Reading, Pseudoword Decoding, and Reading Comprehension subtest AEs are provided in Table 2 for comparison, along with the corresponding nonparametric descriptive statistics for SSs on the same measures. For children aged 9 years 0 months–9 years 3 months, the AE range corresponding to SSs ± 1 *SD* from the general population mean (85–114) is 7.67–11.00 years for Word Reading, 7.00–14.00 years for Pseudoword Decoding, and 7.00–16.00 years for Reading Comprehension. For each of the three reading measures, the AE at the 75th percentile for the present participants was the same as the AE at the 16th percentile for same-aged TD children.

**Table 2:** Descriptive statistics for WIAT-III age equivalents and standard scores

Variable	Median	Interquartile Range	Range
Age equivalent			
Word Reading	6.67 yrs	6.00–7.67 yrs	< 6.00^a^–10.00 yrs
Pseudoword Decoding	6.33 yrs	< 6.00^a^–7.00 yrs	< 6.00^a^–11.00 yrs
Reading Comprehension	6.33 yrs	6.00–7.00 yrs	< 6.00^a^–13.00 yrs
Standard score			
Word Reading	72.50	63.75–83.50	50^b^–108
Pseudoword Decoding	73.00	63.00–84.00	59^c^–107
Basic Reading Composite	73.00	63.00–83.25	52^b^–106
Reading Comprehension	69.00	57.75–82.25	40^d^–110

*N* = 70. Age equivalents are not available for Basic Reading Composite. WIAT-III = Wechsler Individual Achievement Test-III

^a^Lowest possible age equivalent

^b^Lowest possible standard score for children aged 9 years 8 months–9 years 11 months

^c^Lowest possible standard score for children aged 9 years 4 months–9 years 11 months

^d^Lowest possible standard score

To compare children’s word reading abilities to their reading comprehension abilities, a dependent *t*-test comparing WIAT-III Basic Reading SS to WIAT-III Reading Comprehension SS was conducted. For this analysis, children with Reading Comprehension SSs < 53 (the lowest possible Basic Reading Composite SS for children aged 9 years 0 months–9 years 3 months) were assigned a SS of 53 so that the floor on each measure would be the same. This procedure increased the mean Reading Comprehension SS slightly to 70.41 (*SD* = 15.15). Mean Basic Reading SS was significantly higher than mean Reading Comprehension SS, *t*(69) = 3.59, *p* = .001, *d* = 0.23.

To compare children’s real-word reading abilities to their pseudoword reading abilities, a dependent *t*-test comparing WIAT-III Word Reading SS (floored at 60, the lowest possible Pseudoword Decoding SS for children aged 9 years 0 months–9 years 3 months) to WIAT-III Pseudoword Decoding SS was conducted. Mean SSs were 75.03 (*SD* = 12.85) for Word Reading and 73.37 (*SD* = 11.56) for Pseudoword Decoding. The difference was not statistically significant, *t*(69) = 1.52, *p* = .134, *d* = 0.09.

### Multiple regression analyses

Maternal Education level was not significantly correlated with either Basic Reading SS (*r* = .11, *p* = .366) or Reading Comprehension SS (*r* = .18, *p* = .133). Therefore, Maternal Education was not included in the multiple regression models. All assumptions of multiple linear regression analyses were met. Cohen’s *f*^2^ was used to measure effect size (0.02 = small effect, 0.15 = medium, 0.35 = large; Cohen, 1988). Pearson correlations (α = .01) among the variables included in the regression analyses are reported in Table 3. All correlations except for the one between Reading Instruction Method and Spatial SS were statistically significant.

**Table 3:** Bivariate correlations among the measures included in the regression analyses

Measure	2	3	4	5	6	7	8	9	10
1. Vocabulary SS	.67**	.51**	.70**	.39*	.68**	.85**	.37*	.52**	.63**
2. Nonverbal Reasoning SS		.62**	.59**	.58**	.62**	.67**	.57**	.65**	.73**
3. Spatial SS			.52**	.47**	.50**	.57**	.27	.50**	.54**
4. Phonological skills T				.42**	.64**	.68**	.47**	.64**	.69**
5. Rapid Naming T					.44**	.41**	.41**	.49**	.48**
6. Verbal working memory T						.72**	.49**	.64**	.68**
7. Listening comprehension SS							.50**	.65**	.75**
8. Reading instruction method								.82**	.70**
9. Basic Reading Composite SS									.85**
10. Reading Comprehension SS									

*N* = 70. SS = standard score; T = T-score

* *p* < .01. ** *p* < .001

#### Concurrent predictors of Basic Reading SS

To identify significant concurrent predictors of single-word reading accuracy, we began by computing a multiple regression model that included the two independent variables most consistently associated with decoding: Reading Instruction Method and Phonological Skills (Model 1). This model explained a large amount of the variance in Basic Reading SS, *R*^2^ = .76, adjusted *R*^2^ = .75, *F*(2, 67) = 103.98, *p* < .001 (Table 4). The effect size was large for both Reading Instruction Method and Phonological Skills. After controlling for the effect of Phonological Skills T, Phonics instruction resulted in a Basic Reading SS 16.71 points higher than Other reading instruction approaches. After controlling for Reading Instruction Method, a 1-point increase in Phonological Skills T resulted in a 0.45-point increase in Basic Reading SS.

**Table 4:** Multiple regression analyses predicting concurrent WIAT-III Basic Reading Composite standard score

Predictor	*B*	*t*	*p*-value	95% CI for *B*	Semi-partial *r*	Cohen’s *f*^2^
Model 1						
Constant	66.11	60.34	< .001	[63.92, 68.29]		
Reading instruction method	16.71	9.69	< .001	[13.27, 20.15]	.58	1.40
Phonological skills T	0.45	4.96	< .001	[0.27, 0.63]	.30	0.36
*R*^2^ = .76, adjusted *R*^2^ = .75, *F*(2, 67) = 103.98, *p* < .001						
Model 2 (final model)						
Constant	66.70	58.92	< .001	[64.43, 68.96]		
Reading instruction method	15.42	8.09	< .001	[11.61, 19.23]	.46	1.05
Phonological skills T	0.26	2.22	.030	[0.03, 0.49]	.13	0.08
Vocabulary SS	− 0.04	− 0.45	.652	[− 0.23, 0.15]	− .03	< 0.01
Nonverbal Reasoning SS	< 0.01	0.03	.978	[− 0.20, 0.21]	< .01	< 0.01
Spatial SS	0.15	2.06	.044	[< 0.01, 0.29]	.12	0.06
Verbal working memory T	0.16	1.74	.086	[− 0.02, 0.34]	.10	0.06
Rapid Naming T	0.05	0.43	.671	[− 0.20, 0.31]	.02	< 0.01
*R*^2^ = .80, *R*^2^ change = .04, adjusted *R*^2^ = .77, *F*(5, 62) = 2.44, *p* = .044, Cohen’s *f*^2^ = 0.20						

*N* = 70. All continuous independent variables were centered on the sample mean. WIAT-III = Wechsler Individual Achievement Test-III; CI = confidence interval; SS = standard score; T = T-score

To determine if other independent variables that previously have been identified in some studies as having a significant effect on single-word reading also contributed significantly to Basic Reading SS beyond the effects of Reading Instruction Method and Phonological Skills T, we performed an additional multiple regression analysis (Model 2) in which five independent variables (Vocabulary SS, Nonverbal Reasoning SS, Spatial SS, Verbal Working Memory T, and Rapid Naming T) were added to Model 1. Model 2 (the final model) accounted for significantly more variance in Basic Reading SS than did Model 1, *R*^2^ = .80, *R*^2^ change = .04, adjusted *R*^2^ = .77, *F*(5, 62) = 2.44, *p* = .044, Cohen’s *f*^2^ = 0.20. Reading Instruction Method (large effect), Phonological Skills T (small effect), and Spatial SS (small effect) made significant independent contributions to the variance in Basic Reading SS (Table 4). Analyses excluding children who were not able to read at least one of the real words and/or pseudowords and analyses with Word Reading SS and Pseudoword Decoding SS as dependent variables yielded a similar pattern of findings (Supplemental Materials).

#### Concurrent predictors of Reading Comprehension SS

To identify significant predictors of reading comprehension ability, we first conducted a multiple regression that included the two independent variables that most consistently have been associated with reading comprehension: Basic Reading Composite and Listening Comprehension (Model 1, the final model). This model accounted for a large amount of variance in Reading Comprehension SS, *R*^2^ = .79, adjusted *R*^2^ = .79, *F*(2, 67) = 127.06, *p* < .001 (Table 5). The effect size was large for Basic Reading SS and medium for Listening Comprehension SS. After controlling for the effect of Listening Comprehension SS, a 1-point increase in Basic Reading SS resulted in a 0.90-point increase in Reading Comprehension SS. After controlling for the effect of Basic Reading SS, a 1-point increase in Listening Comprehension SS resulted in a 0.43-point increase in Reading Comprehension SS.

**Table 5:** Multiple regression analysis predicting concurrent WIAT-III Reading Comprehension standard score

Predictor	*B*	*t*	*p*-value	95% CI for *B*	semi-partial *r*	Cohen’s *f*^2^
Model 1 (final model)						
Constant	68.23	68.07	< .001	[66.23, 70.23]		
Basic Reading Composite SS	0.90	8.64	< .001	[0.70, 1.11]	.48	1.11
Listening comprehension SS	0.43	4.55	< .001	[0.24, 0.62]	.25	0.31
*R*^2^ = .79, adjusted *R*^2^ = .79, *F*(2, 67) = 127.06, *p* < .001						

*N* = 70. All continuous independent variables were centered on the sample mean. WIAT-III = Wechsler Individual Achievement Test-III; CI = confidence interval; SS = standard score

We performed an additional multiple regression to evaluate if Nonverbal Reasoning SS, Verbal Working Memory T, and Rapid Naming T contributed significant unique variance to reading comprehension beyond the effects of Basic Reading SS and Listening Comprehension SS. Addition of these independent variables did not result in a significant increase in the amount of variance explained in Reading Comprehension SS, *R*^2^ = .81, *R*^2^ change = .02 (*p* = .106), adjusted *R*^2^ = .80, *F*(3, 64) = 2.13, *p* = .106, Cohen’s *f*^2^ = 0.10. Analyses excluding children who earned a raw score of 0 on the WIAT-III Reading Comprehension subtest yielded the same findings (Supplemental Materials).

## Discussion

The present study examined the variability of reading ability and its concurrent predictors in an unprecedentedly large sample of 9-year-old children with WS. In keeping with previous research, reading ability varied widely, ranging from inability to read any words to reading comprehension at age level. As discussed below, the major factors that have been found to affect reading acquisition by TD children also were the major concurrent predictors of reading ability for this sample of children with WS.

### Concurrent predictors of Basic Reading Composite standard score in Williams syndrome

Among the factors we considered as potential correlates of word reading and decoding ability, reading instruction method emerged as a particularly strong concurrent predictor. As has been reported for TD children (e.g., National Reading Panel, 2000), children with WS who were learning to read by a systematic phonics approach earned significantly higher Basic Reading SSs than those learning to read by other reading instruction approaches. As Basic Reading SS was the strongest concurrent predictor of Reading Comprehension SS, the effect of systematic phonics instruction on word reading ability extended to reading comprehension ability. These findings are consistent with those reported in Mervis (2009) for the performance of 9–17-year-olds with WS on the WIAT-II. The effect size for reading instruction method in the present study is larger than that reported by the National Reading Panel (2000) for first graders at risk for future reading problems, providing further evidence of the value of systematic phonics instruction for children with WS.

Our results also corroborate previous findings of a strong association between phonological skills and decoding ability in individuals with WS (e.g., Brawn et al., 2018; Laing et al., 2001). Furthermore, similar to prior findings for TD children (Lafrance & Gottardo, 2005), phonological skills contributed significantly to Basic Reading SS even after controlling for the effects of several other factors previously associated with word reading ability, namely vocabulary, nonverbal reasoning, visual-spatial, verbal working memory, and rapid naming skills. After taking into account the contribution of reading instruction method and phonological processing, the only other variable that also made a significant independent contribution to Basic Reading SS was visual-spatial ability, a skill more rarely studied in relation to reading ability in the general population but that correlates strongly with reading ability in individuals with WS (e.g., Laing et al., 2001).

The two skills that were significant concurrent predictors of reading ability beyond the contribution of reading instruction method are phonological processing, one of the strongest abilities for individuals with WS, and visual-spatial ability, their weakest ability. The confluence of good phonological skills and very weak visual-spatial skills may help explain the large effect found for systematic phonics instruction in the present study. Whereas children with WS can take advantage of their relatively good phonological skills when learning to read by a systematic phonics approach, learning to read by a whole word or whole language approach might be particularly challenging. For example, it is possible that these children's very limited visual-spatial skills interfere with learning to differentiate letter shapes and/or mastering the order of letters in the spelling of words (Dessalegn et al., 2013). Explicit phonics instruction might help counteract some of these negative effects. In particular, learning to read by phonological recoding at the level of grapheme-phoneme correspondences obligatorily draws learners’ attention to the identity and sequence of letters in the spelling of words (Ehri, 2005; see also Share, 1995). Furthermore, as Ehri (2005; Cardoso-Martins & Ehri, 2014) has argued, grapheme-phoneme mappings serve to secure the spellings of individual words in memory and thus provide learners with a powerful mnemonic mechanism for learning the spellings of words and their constituent letters.

Given the large individual differences in the language and cognitive skills evaluated in the present study (Table 1), it may seem surprising that their contribution to word reading and decoding was not significant after controlling for reading instruction method and phonological skills. In light of this possible concern, we compared the magnitude of the correlation between Basic Reading SS and reading instruction method (*r* = .82) to that between Basic Reading SS and DAS-II GCA (*r* = .67). This difference was statistically significant, *z* = 2.22, *p* = .013. Furthermore, Basic Reading SS remained strongly correlated with reading instruction method after controlling for GCA (partial *r* = .75, *p* < .001). In contrast, after controlling for reading instruction method, GCA was only weakly correlated with Basic Reading SS (partial *r* = .27, *p* = .023). These results suggest that Stuebing et al.’s (2009) conclusion that IQ accounts for only a small amount of unique variance in predicting struggling readers’ response to reading instruction can be extended to children with WS. Additional research is needed to examine the extent to which these findings can be extended to individuals with other neurodevelopmental disorders associated with mild to moderate ID.

### Concurrent predictors of reading comprehension in Williams syndrome

Individual differences in decoding and listening comprehension accounted for 79% of the variance in reading comprehension among our participants with WS, a result akin to what has been reported for TD children (Language and Reading Research Consortium & Chiu, 2018). As would be expected given the relatively limited word reading skills of the children with WS in the present study, although both factors accounted for a significant amount of unique variance in Reading Comprehension SS, the effect of decoding was considerably stronger than the effect of listening comprehension. Further studies including older children and adolescents with WS are needed to determine if, as documented for the general population (see García & Cain, 2014, for a meta-analysis), listening comprehension becomes a more important predictor of reading comprehension once children acquire more efficient word reading skills.

As reported in previous studies (Laing et al., 2001; Mervis, 2009), single-word reading was significantly stronger than reading comprehension, a result that is consistent with the cognitive phenotype of individuals with WS. Despite their relatively strong phonological skills and concrete vocabulary, children with WS typically have mild to moderate difficulties in both cognitive (e.g., verbal working memory, comprehension monitoring) and more conceptual language skills (e.g., relational concepts, receptive grammar, discourse, inference-making) (Mervis, 2009). Given the importance of these skills to reading comprehension (Oakhill et al., 2019), research examining how to foster their development in children with WS is needed.

### Limitations and future directions

The results of the present study should be interpreted in the context of certain limitations. One limitation is that the classification of reading instruction method was not based on direct observation. Given the rarity of WS and therefore the need to include participants who resided across a very wide geographical area in order to obtain a substantial number of same-aged children, this limitation was inevitable. To address this limitation, the first author used all available information, including direct interviews with parents and reading instructors, to classify the primary approach to teaching reading in the classroom in which the children received all or most of their reading instruction. Despite efforts to enroll a diverse sample, most of the participants were White non-Hispanic and the majority of the participants’ mothers had completed at least a bachelor degree. Future research with more diverse samples would be valuable.

The cross-sectional nature and correlational design of the present study do not allow us to draw conclusions about causality. Furthermore, as all of the participants were 9 years old, the study does not provide information regarding the developmental trajectory of reading abilities in individuals with WS. At the same time, focusing on a substantial sample of children who were within 12 months of each other in chronological age allowed us to obtain a detailed understanding of both the central tendencies and the variability in word reading and reading comprehension among children with WS of a specific age, as well as the predictors of this variability. Large-sample cross-sectional studies including a broad age range of school-aged children and adolescents are needed to determine the generalizability of the present findings for individuals with WS of other ages. Longitudinal studies in which reading abilities at one age are predicted from hypothesized predictor abilities measured at a younger age are crucial for beginning to address causal relations in the development of reading abilities in individuals with WS.

## Conclusions

Reading ability in 9-year-olds with WS is characterized by great variability, ranging from inability to read any words to reading and comprehending written material at age level. The major factors that affect early reading ability in TD children, namely systematic phonics instruction, phonological skills, and listening comprehension, also are major concurrent predictors of reading ability in WS. The educational implications of these findings are clear: Reading instruction for children with WS should be based on a systematic phonics approach. As Kilpatrick and O’Brien (2019) have argued convincingly, the most effective reading programs combine systematic phonics with extensive phoneme awareness training that includes instruction in phoneme manipulation. Moats (2019) provides an outstanding overview of state-of-the-art systematic phonics instruction within a structured language instruction format that is likely to be effective for children with WS, especially if advanced phoneme awareness (including phoneme manipulation) is incorporated. At the same time, given the weaknesses of children with WS in broader, more conceptual, language skills, it is crucial that reading instruction also incorporate an oral language (listening comprehension) component to allow individuals with WS to take full advantage of their reading skills to advance their academic knowledge, satisfy their curiosity, and read for pleasure.

## Supplementary Information

Below is the link to the electronic supplementary material.

Supplementary file1 (PDF 139 kb)
