# Supplementary material for: Concurrent predictors of word reading and reading comprehension for 9-year-olds with Williams syndrome
Source: Read Writ. 2021 Jul 3;35(2):377–97. doi: 10.1007/s11145-021-10163-4 (PMC8827302; doi:10.1007/s11145-021-10163-4)
Supplement: Supplementary file 1 — Supplementary file1 (PDF 139 kb) [file 11145_2021_10163_MOESM1_ESM.pdf]

Supplemental Materials

Results

Regression Analyses Excluding Participants with Raw Scores of 0 on the WIAT-III Word Reading and/or Pseudoword Decoding Subtests

In some of the previous studies addressing the single-word reading ability of individuals with Williams syndrome (WS), participants who were not able to read any of the real words on the assessment were excluded from the analyses. In other studies, participants who were not able to read any of the pseudowords were excluded. To provide parallel analyses, two additional multiple regressions including Reading Instruction Method and Phonological Skills as independent variables and Basic Reading Composite SS as the dependent variable were conducted. In the present sample, three of the 70 participants (4.29%) did not read any of the real words correctly and 11 (15.71%) – including the three who did not read any of the real words correctly – did not read any of the pseudowords correctly. All 11 children were in the Other group. When the three children who did not read any real words correctly were excluded, Model 1 $R^2 = .73$, adjusted $R^2 = .73$, $F(2,64) = 88.50$, $p < .001$. When the 11 children who did not read any pseudowords correctly were excluded, Model 1 $R^2 = .70$, adjusted $R^2 = .69$, $F(2,56) = 66.40$, $p < .001$. For both regressions, there were significant effects of Reading Instruction Method ($p < .001$) and Phonological Skills T ($p < .001$). Model 2 (in which five independent variables – Vocabulary SS, Nonverbal Reasoning SS, Spatial SS, Verbal Working Memory T, Rapid Naming T – were added to Model 1) did not account for significantly more variance in Basic Reading Composite SS than Model 1 for either of these samples (ps > .10).

Regression analyses for WIAT-III Word Reading and Pseudoword Decoding Subtests

Two additional multiple regression analyses with Reading Instruction Method and Phonological Skills T as the independent variables were conducted, one for each of the subtests included in the WIAT-III Basic Reading Composite. For the regression with Word Reading SS as the dependent variable, $R^2 = .67$, adjusted $R^2 = .66$, $F(2,67) = 67.84$, $p < .001$. The effects of both Reading Instruction Method ($p < .001$, semi-partial $r = .56$) and Phonological Skills T ($p = .001$, semi-partial $r = .27$) were significant. For the regression with Pseudoword Decoding SS as the dependent variable, $R^2 = .74$, adjusted $R^2 = .73$, $F(2,67) = 96.41$, $p < .001$. The effects of Reading Instruction Method ($p < .001$, semi-partial $r = .59$) and Phonological Skills T ($p < .001$, semi-partial $r = .28$) were both significant.

Regression Analyses Excluding Participants with Raw Scores of 0 on the WIAT-III Reading Comprehension Subtest

Eight children (all in the Other group) earned a raw score of 0 on the WIAT-III Reading Comprehension subtest. These included the three children who had a raw score of 0 on both the Word Reading and Pseudoword Decoding subtests, two children who had a raw score > 0 on Word Reading but a raw score of 0 on Pseudoword Decoding, and three children who had raw scores > 0 on both Word Reading and Pseudoword Decoding. When the Model 1 regression analysis predicting Reading Comprehension SS was repeated with these children excluded, Model 1 continued to explain a large amount of the variance in Reading Comprehension SS, $R^2 = .75$, adjusted $R^2 = .74$, $F(2,59) = 88.38$, $p < .001$, with significant effects of both Basic Reading Composite SS ($p < .001$, semi-partial $r = .50$) and Listening Comprehension SS ($p < .001$, semi-partial $r = .28$). Model 2 (in which three independent variables – Nonverbal Reasoning SS,

Verbal Working Memory T, Rapid Naming T – were added to Model 1) did not account for significantly more variance in Reading Comprehension SS than Model 1 (*p* = .072).
